# Understanding the Impact of Nitrogen Availability: A Limiting Factor for Enhancing Fucoxanthin Productivity in Microalgae Cultivation

**DOI:** 10.3390/md22020093

**Published:** 2024-02-18

**Authors:** To Quyen Truong, Yun Ji Park, Jessica Winarto, Phuong Kim Huynh, Jinyoung Moon, Yeong Bin Choi, Dae-Geun Song, Song Yi Koo, Sang Min Kim

**Affiliations:** 1 Division of Bio-Medical Science & Technology, Korea Institute of Science and Technology (KIST) School, University of Science and Technology, Seoul 02792, Republic of Korea; ttquyen_vn@kist.re.kr (T.Q.T.); jessicawinarto@kist.re.kr (J.W.); 623024@kist.re.kr (P.K.H.); 2 Smart Farm Research Center, KIST Gangneung Institute of Natural Products, Gangneung 25451, Republic of Korea; pyj@kist.re.kr (Y.J.P.); jy77mun@kist.re.kr (J.M.); choiyeongbin@kist.re.kr (Y.B.C.); 3 Natural Product Informatics Research Center, KIST Gangneung Institute of Natural Products, Gangneung 25451, Republic of Korea; dsong82@kist.re.kr (D.-G.S.); ninesong2@kist.re.kr (S.Y.K.)

**Keywords:** microalgae, fucoxanthin, nitrogen availability, *Phaeodactylum tricornutum*, FCP protein

## Abstract

This study aimed to investigate the regulation of fucoxanthin (FX) biosynthesis under various nitrogen conditions to optimize FX productivity in *Phaeodactylum tricornutum*. Apart from light, nitrogen availability significantly affects the FX production of microalgae; however, the underlying mechanism remains unclear. In batch culture, *P. tricornutum* was cultivated with normal (NN, 0.882 mM sodium nitrate), limited (LN, 0.22 mM), and high (HN, 8.82 mM) initial nitrogen concentrations in f/2 medium. Microalgal growth and photosynthetic pigment production were examined, and day 5 samples were subjected to fucoxanthin–chlorophyll *a/c*-binding protein (FCP) proteomic and transcriptomic analyses. The result demonstrated that HN promoted FX productivity by extending the exponential growth phase for higher biomass and FX accumulation stage (P1), showing a continuous increase in FX accumulation on day 6. Augmented FX biosynthesis via the upregulation of carotenogenesis could be primarily attributed to enhanced FCP formation in the thylakoid membrane. Key proteins, such as LHC3/4, LHCF8, LHCF5, and LHCF10, and key genes, such as *PtPSY*, *PtPDS*, and *PtVDE,* were upregulated under nitrogen repletion. Finally, the combination of low light and HN prolonged the P1 stage to day 10, resulting in maximal FX productivity to 9.82 ± 0.56 mg/L/day, demonstrating an effective strategy for enhancing FX production in microalgae cultivation.

## 1. Introduction

Fucoxanthin (FX), a pigment derived from marine algae, has been shown in in vitro and in vivo studies to possess various health-promoting effects, such as anti-cancer, anti-diabetic, anti-inflammatory, anti-obesity, and antioxidant properties [1]. FX exhibits potent capabilities in scavenging free radicals, such as singlet oxygen, hydrogen peroxide, and superoxide anions, thereby alleviating oxidative stress [2]. FX reduces the production of proinflammatory cytokines responsible for cellular inflammation, including TNF-α, IL-1β, IL-6, and IFN-γ [3]. Furthermore, the anti-cancer effects of FX and its deacetylated product fucoxanthinol occur via cell cycle arrest, apoptosis induction, and angiogenesis inhibition [4].

FX stands out as the most abundant xanthophyll in nature, constituting approximately 10% of all naturally occurring carotenoids. The FX pigment is present in the chloroplasts of brown macroalgae, such as *Laminaria japonica*, *Sargassum fusiforme*, and *Undaria pinnatifida,* as well as microalgae, including *Isochrysis* aff. *galbana*, *Tisochrysis lutea*, *Phaeodactylum tricornutum*, and *Odontella aurita* [5]. Currently, brown macroalgae are the major source of FX supply for commercial production despite their low cellular FX content reported between 0.02 and 4.96 mg/g fresh weight (FW) [6]. In comparison with macroalgae, microalgae could produce considerably higher FX content ranging from 5.4 to 79.4 mg/g dried weight in recent studies [6]. Notably, marine diatoms, such as *P. tricornutum,* have been recognized for their potential as cell factories of high-value products, including chrysolaminarin, eicosapentaenoic acid, and FX [7]. As a primary light-harvesting pigment, FX biosynthesis is essential for both photosynthesis and microalgal growth. FX plays a crucial role in transferring light photons to chlorophylls, which is particularly vital for optimizing photosynthetic efficiency to sustain growth and development under suboptimal light conditions [8].

Owing to the health benefits and economic significance of FX, its biosynthesis has been the subject of extensive research, especially in diatoms [9]. Geranylgeranyl pyrophosphate (GGPP) is the final product of the methylerythritol 4-phosphate (MEP) pathway and serves as a precursor for carotenogenesis, leading to FX synthesis. This process is initiated with the condensation of GGPP catalyzed by phytoene synthase (PSY). Putative enzymes regulating subsequent steps encompass phytoene desaturases (PDS), carotenoid isomerase (CRTISO), ζ-carotene desaturases (ZDS), lycopene *β*-cyclase (LYCb), zeaxanthin epoxidases (ZEP), violaxanthin de-epoxidases (VDE and VDL), and *VDE* related (VDR). Noteworthy findings include the positive effects of the overexpression of *PSY* and *VDR/VDE/ZEP-3* on FX accumulation in *P. tricornutum* [10,11]. Moreover, previous findings have indicated that the overexpression of specific genes regulating the MEP pathway considerably enhances FX biosynthesis. For example, 1-deoxy-D-xylulose 5-phosphate synthase (*DXS*) overexpression could enhance up to 2.4-fold FX content [12], while 4- (cytidine 5′-diphospho)-2-C-methyl-D-erythritol kinase (*CMK*) overexpression could double the FX content in *P. tricornutum* [13].

Most photosynthetic pigments are intricately associated with antenna proteins and form light-harvesting complexes. In diatoms, FX molecules are attached to antenna proteins known as fucoxanthin–chlorophyll *a*/*c*-binding proteins (FCPs). FCP complexes are embedded in the thylakoid membrane, which is responsible for light harvesting and photoprotection [14]. The monomer structure of FCP encompasses antenna proteins bound to photosynthetic and photoprotective pigment molecules, including FX, chlorophylls *a* and *c*, diadinoxanthin, and diatoxanthin [14]. The identified FCP antenna proteins are categorized into three major LHC classes: LHCFs (major FCP antenna proteins), LHCRs (red alga-like proteins), and LHCXs (related to the LhcSRs in *Chlamydomonas reinhardtii*).

Previous findings indicate that various environmental and nutrient factors influence FX production by microalgae [15]. Among these, light had the most profound effect on the cellular FX content and FX productivity [16]. In particular, reduced light intensity and blue light likely increased FX concentration, whereas excessive light intensity and red light diminished FX accumulation [17,18]. Our previous study underscored the interdependence of FCP and FX biosynthesis in *P. tricornutum* in response to fluctuating light conditions [17]. Moreover, the correlation between FX and FCP has been subtly indicated in previous bioengineering reports [19,20,21]. The simultaneous knockout of six *LHCF* genes, including *LHCF1*, *2*, *3*, *4*, *5*, and *11*, was performed by Sharma et al. [19]. The knock-out of *LHCF1*, *LHCF2*, and *LHCF5* in *P. tricornutum* resulted in a lighter brown color, whereas mutations in *LHCF3* and *LHCF4* resulted in a green hue. In line with these observations, the mutants produced less FX pigment than the wild type.

Apart from light, nitrogen also plays a substantial role in FX production because nitrogen, a vital macronutrient found in the culture medium, is essential for microalgal growth and development owing to its presence in nucleic acid and protein-building blocks. Microalgae can use both organic and inorganic nitrogen sources, such as ammonium, nitrate, tryptone, and urea. Many findings have revealed that different nitrogen concentrations can extensively alter the growth, biochemical composition, and synthesis of bioactive compounds, such as fatty acids and pigments [22,23]. In particular, the influence of nitrogen availability on fatty acids and FX, the two most abundant high-value compounds found in diatoms, has been studied extensively [24,25]. Previous studies have indicated that nitrogen limitation promotes lipid accumulation and reduces FX production. Several studies have employed different approaches, including different nitrogen sources and regimes, to improve FX production from microalgae cultivation [18,26,27]. Despite the wealth of quantitative results regarding FX production under nitrogen fluctuation, the regulatory mechanisms governing the impact of nitrogen availability on FX biosynthesis remain elusive.

Given the limited information, this systematic investigation aimed to elucidate the impact of nitrogen availability on the regulation of FX biosynthesis in diatoms and apply this information to enhance FX productivity. In this study, the robust model organism *P. tricornutum*, renowned for its adaptability and whole-genome sequence, was used to explore the regulatory mechanism of FX biosynthesis through the effect of nitrogen availability on not only carotenogenesis but also FCP biosynthesis. Briefly, *P. tricornutum* UTEX 646 was cultured with three initial nitrogen concentrations, ranging from low to high. After observing the effect of nitrogen availability on the growth, biomass, and pigment production of *P. tricornutum*, the expression of FCP proteins and key genes involved in related pathways was investigated in day 5 samples when *P. tricornutum* was at different growth stages, depending on nitrogen availability in the media. Firstly, opposite effects of nitrogen depletion and repletion on FX accumulation have been consistently observed. Secondly, our results specifically underscore the correlation between FCP formation and FX biosynthesis in response to nitrogen fluctuation. Subsequently, the up/downregulation of genes involved in nitrogen uptake and assimilation, FCP formation, and carotenogenesis was systematically reported for the first time. For industrial applications, the importance of nitrogen repletion in FX production was employed to establish an integrated approach that incorporated well-known factors favoring FX biosynthesis, significantly improving FX productivity. This research not only expands our understanding of FX biosynthesis but also holds potential implications for enhancing FX productivity in microalgae for further applications in diverse fields, notably in the pharmaceutical and food industries.

## 2. Results

### 2.1. Effect of Nitrogen Availability on Various Physiological Characteristics of Phaeodactylum tricornutum

**Figure 1:**
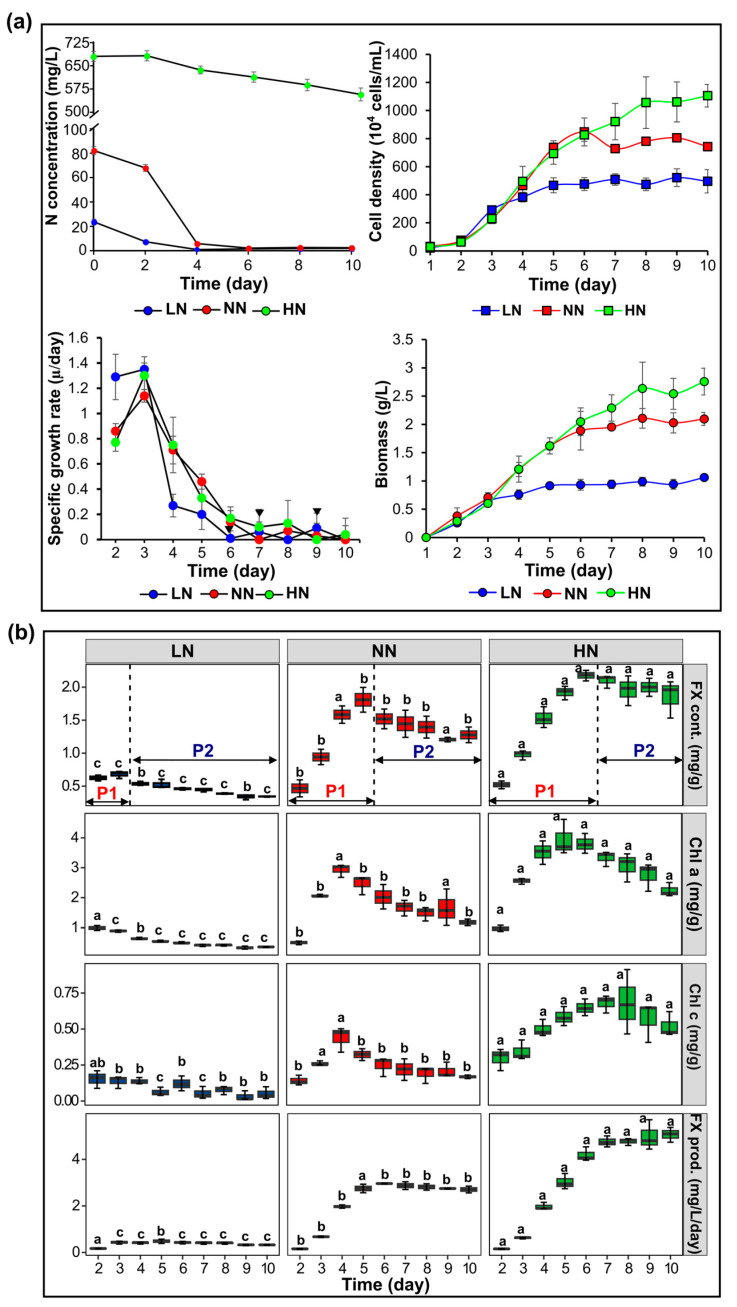
(**a**) Nitrogen (N) concentration available in the culture medium, cell density (×10^4^ cells/mL), specific growth rate, biomass concentration (g/L), and (**b**) content (mg/g FW) of photosynthetic pigments, including fucoxanthin (FX), chlorophylls (Chl) *a* and *c*, and FX productivity of *Phaeodactylum tricornutum* microalgae cultured at different initial nitrogen concentrations [limited nitrogen (LN), normal nitrogen (NN), and high nitrogen (HN)]. Black triangles mark the time when the specific growth rate (µ) was equal to zero in LN, NN, and HN. The dashed line divides the two stages, P1 and P2, of fucoxanthin accumulation in *P. tricornutum*. Different letters denote the significant difference amongst the three groups analyzed with one-way ANOVA followed by Tukey’s test, *p* < 0.05. cont., content; prod., productivity.

*P. tricornutum* was cultivated at varying nitrogen conditions, corresponding to different sodium nitrate concentrations in the f/2 medium, including limited, 0.22 mM, normal, 0.882 mM, and high, 8.82 mM, for 10 days until it reached the stationary phase. Figure 1a shows the growth performance, biomass production, and nitrogen consumption of *P. tricornutum* under different nitrogen conditions. Concurrently, nitrogen uptake by the growing microalgae resulted in a gradual reduction in the nitrogen concentration within the culture medium over time. Consequently, *P. tricornutum* grown in the limited (LN) and normal (NN) initial nitrogen concentrations likely reached a nitrogen-depleted state on days 4 and 6, respectively. However, the high nitrogen concentration available in the culture media of high initial nitrogen (HN) samples was maintained throughout the cultivation period. The growth and biomass production of *P. tricornutum* varied with initial nitrogen concentrations. Compared with NN, LN significantly lowered the growth rate and microalgal biomass, whereas HN promoted them (Figure 1a). *P. tricornutum* grown in HN and NN exhibited higher cell density and biomass concentration than those grown in LN. Notably, HN samples achieved significantly higher cell density and biomass with respect to NN samples from day 6 onwards (*p <* 0.05). Finally, the highest cell density obtained from LN, NN, and HN were approximately 5 × 10^6^, 8 × 10^6^, and 1.1 × 10^7^ cells/mL, respectively, while the highest biomass concentration on day 10 was 1.06 ± 0.02, 2.10 ± 0.08, and 2.76 ± 0.24 g/L, respectively. Zero value of the specific growth rate (µ), marking the end of the growth phase, was recorded on days 6, 7, and 9 at LN, NN, and HN conditions, respectively.

As mentioned previously, FX is a protein-bound pigment, recognized for its co-existence together with chlorophyll molecules in the FCP complexes. A highly positive correlation of FX content and chlorophyll *a*, *c* in *P. tricornutum* samples was observed under varying nitrogen conditions (r > 0.9, *p* < 0.5, Figure S1). Figure 1b depicts the accumulation of primary photosynthetic pigments and FX productivity of *P. tricornutum* grown under different initial nitrogen concentrations. Particularly, the highest chlorophyll *a* content (3.94 ± 0.07 mg/g FW) was obtained from HN samples, whereas NN and LN samples contained lower chlorophyll *a* content (2.93 ± 0.02 and 0.99 ± 0.08 mg/g FW, respectively). The highest chlorophyll *c* content obtained from the LN, NN, and HN samples was 0.15 ± 0.06, 0.44 ± 0.09, and 0.68 ± 0.06 mg/g FW, respectively. The accumulation of photosynthetic pigments, specifically FX accumulation, exhibited two distinct stages, P1 and P2, with opposite patterns (Figure 1b). Stage P1 was associated with a continuous increase in FX content, whereas FX accumulation experienced a downward trend during the latter stage, P2. Stage P1 was shortened in LN (3 day) and was extended in the HN condition (6 day). Consistently, HN samples produced the highest FX content at the end of stage P1 and FX productivity (2.18 ± 0.08 mg/g FW and 5.07 ± 0.32 mg/L/day) during stage P2, while LN and NN samples had significantly lower FX content (0.68 ± 0.06 and 1.81 ± 0.19 mg/g FW) and FX productivity (0.48 ± 0.09 and 2.96 ± 0.01 mg/L/day) (*p* < 0.05), respectively.

### 2.2. Semi-Quantification of FCP Complexes in the Thylakoid Membrane of Day 5 Samples

Semi-quantification of FCP complexes in the thylakoid membrane was conducted using *P. tricornutum* samples collected on day 5, where LN, NN, and HN were associated with stationary, late exponential, and mid-exponential phases, respectively (Figure 1a). Analyses of FCP proteomic and photosynthetic pigments in the thylakoid membrane revealed that nitrogen conditions markedly affected FCP abundance in day 5 samples (Table 1, Figure S2). In particular, fourteen proteins from the LHCF family, six proteins from the LHCR family, and one LHCX protein were identified (Table 1). Similar to our previous findings, LHCF3/4 (B7FRW2) was found to be the most abundant component among the identified antenna proteins. The other major protein components included LHCF8 (B7G6Y1), LHCF5 (B7GBK7), LHCF10 (B7G5B6), and LHCF11 (B7GBK6). Compared with LN, HN promoted the enrichment of FCP antenna proteins in the thylakoid membrane, which was consistent with FX production. Aligning with this result, the photosynthetic pigment contents in the total protein of thylakoid membrane fractions isolated from day 5 samples were significantly increased in HN samples (*p* < 0.05) (Figure S2). In particular, chlorophyll and FX contents in the total thylakoid membrane protein obtained from HN samples were ~1.7 and ~5 times higher than those obtained from NN and LN samples.

### 2.3. Transcriptome Analysis of Phaeodactylum tricornutum under Varying Nitrogen Conditions

**Figure 2:**
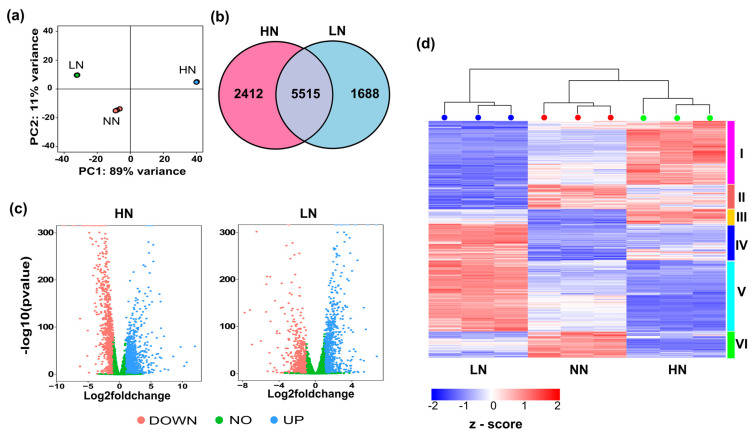
(**a**) Principal component plot of investigated samples. (**b**) Venn diagram of differentially expressed genes in initial high and low nitrate samples (HN and LN). (**c**) Volcano plots of DEGs. (**d**) Z-score heatmap depicting distinct gene clusters in response to different nitrogen conditions. Day 5 samples of *Phaeodactylum tricornutum* microalgae cultured at different initial nitrogen concentrations [limited nitrogen (LN), normal nitrogen (NN), and high nitrogen (HN)] were used for transcriptome analysis.

Transcriptome analysis revealed the distinct characteristics of day 5 *P. tricornutum* samples cultured under different initial nitrogen concentrations (Figure 2a). Analysis of differential gene expression using DESeq2 revealed that 2412 genes were differentially expressed in HN samples, whereas 1688 genes were differentially expressed in LN samples (Figure 2b). Under the HN condition, 3988 genes were upregulated and 3939 genes were significantly downregulated (*p*-adjusted *<* 0.05). Similarly, under the LN condition, 3726 genes were upregulated and 3477 genes were downregulated compared with NN samples (Figure 2c). More than 777 and 459 genes exhibited |log2foldchange| ≥ 2 in day 5 HN and LN samples, respectively. A total of 10,961 genes were identified with a cutoff of <5 and classified into six different clusters by grouping genes exhibiting similar co-expression patterns across various nitrogen conditions (Figure 2d).

The two most abundant clusters, I and V, provided genes exhibiting opposite patterns in HN and LN samples. Specifically, genes in cluster I were mostly upregulated in HN samples and downregulated in LN samples, whereas genes in cluster V were highly expressed in LN samples and downregulated in HN samples. Gene ontology (GO) enrichment and the Kyoto Encyclopedia of Genes and Genomes (KEGG) revealed the top processes and activities in which genes in clusters I and V were enriched (Figures S3 and S4). The most abundant GO terms in biological processes include macromolecules, nitrogen compounds, and proteins. In the cellular component, common processes include the cytoplasm, membrane-bounded organelles, and organelles. Notably, the formation of thylakoids, photosystems, and light-harvesting complexes was found in cluster I, whereas the formation of the ribosome and non-membrane-bounded organelles was observed in cluster V. In terms of molecular function, many genes in clusters I and V were commonly involved in various binding activities, including nucleotide, ion, small molecule, carbohydrate derivative, and anion binding activities. Further, KEGG analysis revealed that the genes in cluster I were enriched in various metabolic pathways, including the biosynthesis of secondary metabolites, glycolysis, carbon metabolism, and the biosynthesis of cofactors. Genes in cluster V were also enriched in various metabolic pathways, such as the biosynthesis of secondary metabolites, ribosomes, oxidative phosphorylation, carbon metabolism, and the biosynthesis of amino acids.

**Figure 3:**
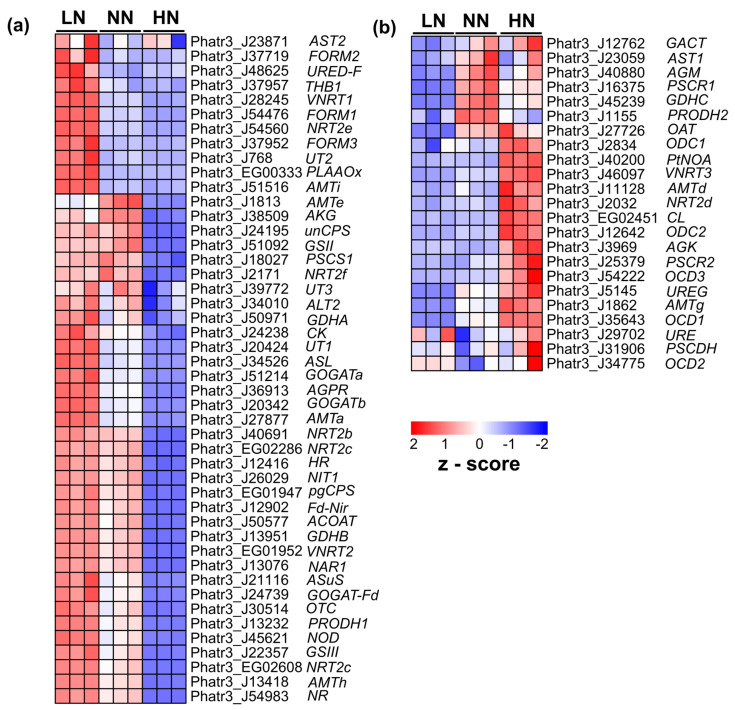
Downregulation (**a**) and upregulation (**b**) of genes involved in nitrogen uptake and assimilation pathways in *Phaeodactylum tricornutum* microalgae cultured at high initial nitrogen (HN) compared to limited nitrogen (LN) and normal nitrogen (NN). Data were retrieved from RNA sequencing analysis of day 5 samples. AAT, aspartate aminotransferase; ACOAT, acetylornithine aminotransferase; AGK, acetyl-1-glutamate kinase; AGPR, N-acetyl-gamma-glutamyl-phosphate reductase; AKG, acetylglutamate kinase; ALT, L-alanine transaminase; AMT, ammonium transporter; ASL, argininosuccinate lyase; ASuS, argininosuccinate hydratase; CK, carbamate kinase; CPS, carbomoyl phosphate synthetase; CYN, cyanate hydratase; Fd-Nir, nitrite reductase; FORM, formamidase; GACT, glutamate N-acetyltransferase; GDH, glutamate dehydrogenase; GLN, glutamine synthase; GLT; NADH-dependent glutamate synthase; GOGAT, glutamate synthase; GSII/III, glutamate–ammonia ligase; HR, hydroxylamine reductase; NAR1, nitrite transporter; NIT, nitrate transporter 2.4; NR, nitrate reductase; NRT, nitrate transporter; OAT, ornithine cyclodeaminase; OCD, ornithine cyclodeaminase; ODC, ornithine decarboxylase; OTC, ornithine transcarbamylase; PLAAOx, periplasmic L-amino acid oxidase; PRODH, methylenetetrahydrofolate reductase family; PSCDH, aldehyde dehydrogenase; PSCR, pyrroline-5-carboxylate (P5C) reductase (P5CR); unCPS, carbamoyl phosphate synthase mitochondrial; URE, urease; UT, urea transporter; VNRT, vacuolar nitrate transporter.

Transcriptome analysis showed that most genes involved in nitrogen uptake and assimilation exhibited increased expression in LN samples but decreased expression in HN samples (Figure 3a). Another group of genes exhibited the opposite expression pattern (Figure 3b). In comparison with NN, genes such as *PtACOAT* (Phatr3_J50577), *PtFd-NiR* (Phatr3_J12902), *PtNAR1* (Phatr3_J13076), *PtNIT1* (Phatr3_J26029), *PtNR* (Phatr3_J54983), *PtNRT* (Phatr3_EG02286 and Phatr3_EG02608), and *PtVNRT2* (Phatr3_EG01952) were sharply downregulated in HN samples with log2foldchange < −5 (Table S1). On day 5, most genes exhibited similar levels of transcript expression in LN and NN samples with | log2foldchange | < 1 (Figure 3, Table S1).

**Figure 4:**
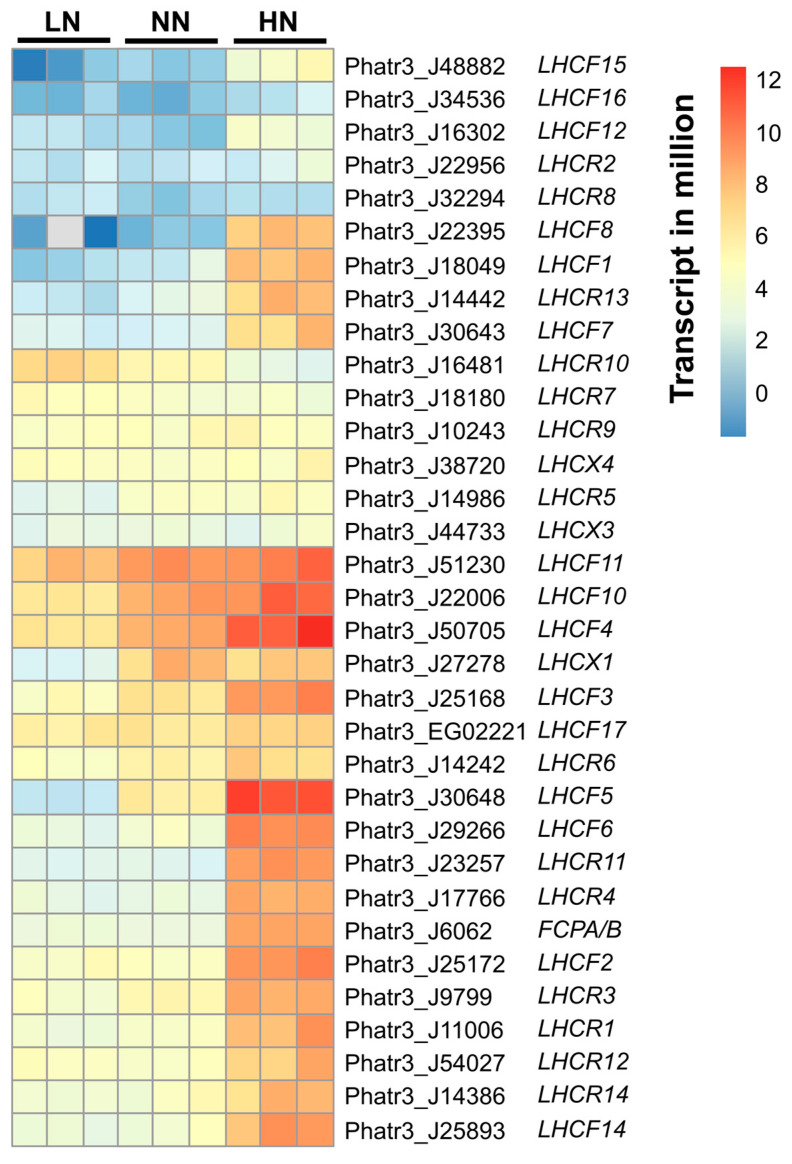
Relative transcript levels of fucoxanthin–chlorophyll *a/c*-binding protein-encoding genes in *Phaeodactylum tricornutum* microalgae cultured at different initial nitrogen concentrations [limited nitrogen (LN), normal nitrogen (NN), and high nitrogen (HN)]. Data were retrieved from RNA sequencing analysis of day 5 samples. LHCFs, main fucoxanthin–chlorophyll *a/c*-binding proteins; LHCRs, red alga-like proteins; LHCXs, related to the LhcSRs in *Chlamydomonas reinhardtii*.

Regarding FCP biosynthesis, transcriptome analysis revealed the marked effect of varying nitrogen conditions on the regulation of *LHC* genes. In this study, sixteen *LHCF*, fourteen *LHCR*, and three *LHCX* gene sequences were identified (Figure 4). Among these, *PtLHCF3* (Phatr3_J25168), *PtLHCF4* (Phatr3_J50705), *PtLHCF5* (Phatr3_J30648), *PtLHCF10* (Phatr3_J22006)*,* and *PtLHCF11* (Phatr3_J51230) had the most abundant transcripts, which is consistent with the results of the FCP proteomic analysis in Table 1. The expression levels of FCP-encoding genes corresponded to nitrogen concentrations. Notably, *PtLHCF1* (Phatr3_J18049), *PtLHCF2* (Phatr3_25172), *PtLHCF5* (Phatr3_J30648), *PtLHCF6* (Phatr3_J29266), *PtLHCF7* (Phatr3_J30643), *PtLHCF8* (Phatr3_J22395), *PtLHCF12* (Phatr3_J16302), *PtLHCF14* (Phatr3_J25893), *PtLHCF15* (Phatr_J48882), *PtLHCR1* (Phatr3_J11006), *PtLHCR4* (Phatr3_J17766), *PtLHCR11* (Phatr3_J23257), *PtLHCR13* (Phatr3_J14442), and *PtFCPA/B* (Phatr3_J6062) were upregulated with log2foldchange > 3 in HN samples (Table S2). Most genes showed comparable expression levels between LN and NN samples, yet *PtLHCF7, PtLHCF15,* and *PtLHCF17* were significantly downregulated with log2foldchange < −1.5 in LN samples on day 5 (*p*-adjusted *<* 0.05, Table S2).

**Figure 5:**
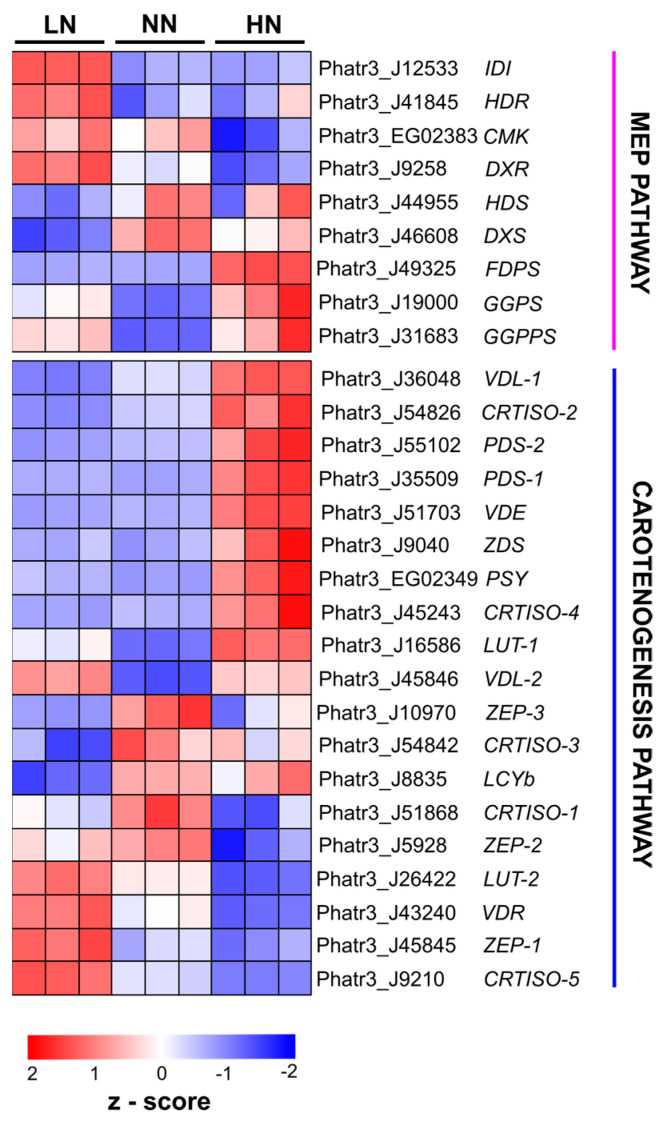
Regulation of genes involving methylerythritol 4-phosphate (MEP) and carotenogenesis pathways toward fucoxanthin biosynthesis in *Phaeodactylum tricornutum* microalgae cultured at different initial nitrogen conditions [limited nitrogen (LN), normal nitrogen (NN), and high nitrogen (HN)]. Data were retrieved from RNA sequencing analysis of samples on day 5. CMK, 4- (cytidine 5′-diphospho)-2-C-methyl-D-erythritol kinase; DXR, 1-deoxy-D-xylulose 5-phosphate reductoisomerase; DXS, 1-deoxy-D-xylulose 5-phosphate synthase; FDPS, farnesyl diphosphate synthase; GGPS, geranylgeranyl diphosphate synthase; GGPPS, geranylgeranyl diphosphate synthase; HDS, 4-hydroxy-3-methylbut-2-enyl diphosphate synthase; HDR, 4-hydroxy-3-methylbut-2-enyl diphosphate reductase; IDI, isopentenyl–diphosphate isomerase; CRTISO, carotenoid isomerase; LUT, lutein-deficient 1-like protein; LYCb, lycopene *β*-cyclase; PDS, phytoene desaturases; PSY, phytoene synthase; VDE, violaxanthin de-epoxidase; VDL, violaxanthin de-epoxidase-like protein, VDR, *VDE* related; ZDS, *ꞵ*-carotene desaturases; ZEP, zeaxanthin epoxidases.

Subsequently, transcriptome analysis revealed differential expression of genes involved in the MEP and carotenogenesis pathways, leading to FX biosynthesis in *P. tricornutum* under varying nitrogen conditions. In comparison to NN, sixteen genes were significantly upregulated in HN samples with log2fodchange > 0.1 (*p*-adjusted *<* 0.05) (Table S3). Particularly, genes including *PtCRTISO-2* (Phatr3_J54826), *PtCRTISO-4* (Phatr3_J45243), *PtFDPS* (Phatr3_J49325), *PtGPPS* (Phatr3_J19000), *PtGGPPS* (Phatr3_J31683), *PtPDS-1* (Phatr3_J35509), *PtPDS*-2 (Phatr3_J55102), *PtPSY* (Phatr3_EG02349), *PtVDE* (Phatr3_J51703), and *PtVDL-2* (Phatr3_J45846) were highly upregulated in HN samples with log2foldchange > 1.5 (Figure 5, Table S3). In contrast, genes including *PtCRTISO-5* (Phatr3_J9210) and *PtVDR* (Phatr3_J43240) were highly downregulated in HN samples on day 5 with a log2foldchange < −1.5. Figure 5 revealed a similar expression pattern of investigated genes under LN and NN conditions. Despite that, fifteen genes were slightly downregulated with log2folchange < 0.1 according to differentially expressed gene analysis (Table S3). Particularly, the expression of *PtPDS-2* and *PtVLD-1* was highly reduced under LN conditions. In contrast, genes like *PtVDE* and *PtPDS-1* were abnormally upregulated in LN.

### 2.4. Combination of Light and Nitrogen Supply to Enhance Fucoxanthin Production

**Figure 6:**
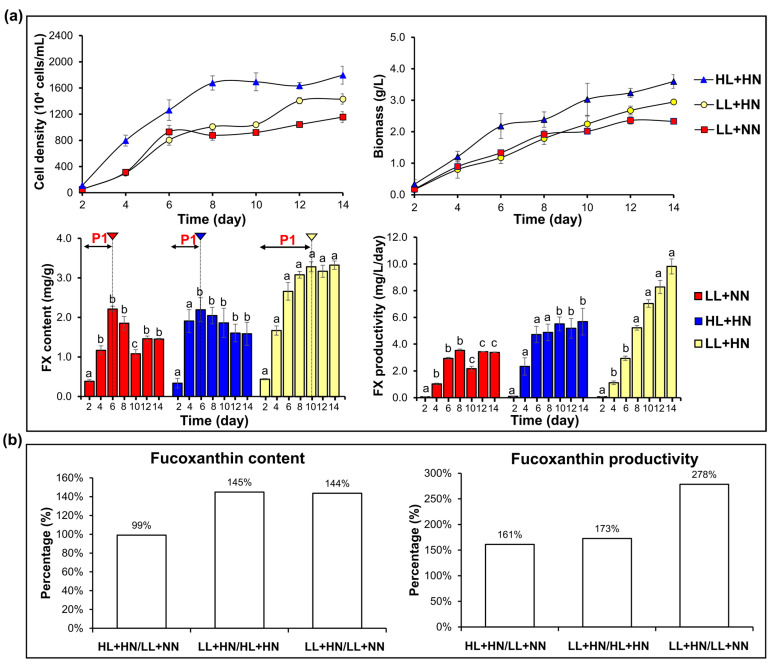
(**a**) Cell density, biomass, fucoxanthin (FX) content, and FX productivity of *Phaeodactylum tricornutum* microalgae cultured at various light and nitrogen conditions, including low light + high initial nitrogen (LL+HN), low light + normal initial nitrogen (LL+NN), high light + high initial nitrogen (HL+HN) for 14 days. (**b**) Comparison between maximum values of FX content and productivity obtained in *P. tricornutum* grown under various light and nitrogen conditions. LL and HL were set at 20 and 100 µmol photons/m^2^/s, respectively. Different letters denote the significant difference amongst three groups analyzed with one-way ANOVA followed by Tukey’s test, *p* < 0.05.

This investigation aimed to explore the potential of various light and nitrogen combinations for improving FX productivity for further applications. To examine the combined effect of the two positive factors (low light and high nitrogen supply) on FX production, three experiments were performed: low light + high initial nitrogen (LL+HN), low light + normal initial nitrogen (LL+NN), and high light + high initial nitrogen (HL+HN). Particularly, HN and NN had initial nitrogen levels of 0.882 and 8.82 mM in the f/2 medium, while HL and LL were set at 100 and 20 µmol photons/m^2^/s of light intensity, respectively. First, *P. tricornutum* exhibited better growth performance and biomass production under higher light intensity (Figure 6a). HL+HN resulted in the highest cell number and biomass, which were 1.8 × 10^7^ cells/mL and 3.60 ± 0.21 mg/L, respectively. Second, elevating the nitrogen concentration consistently prolonged the exponential phase under different light conditions. In particular, *P. tricornutum* reached the plateau phase on days 6, 8, and 12 under the LL+NN, HL+HN, and LL+HN treatments, respectively. The highest biomass values obtained from LL+NN and LL+HN samples were 2.36 ± 0.12 and 2.95 ± 0.04 mg/L, respectively.

*P. tricornutum* produced higher FX content under lower light intensity. Particularly, the highest FX content was 3.33 ± 0.11 mg/g FW in LL+HN samples and 2.19 ± 0.31 mg/g FW in HL+HN samples. *P. tricornutum* grown under LL+NN also had FX content comparable to that of HL+HN. Under LL+NN and HL+HN conditions, FX content remarkably reduced from day 6 onwards. On the contrary, LL+HN treatment noticeably accelerated FX accumulation until day 10 and maintained high FX levels during the latter phase. Thus, the LL+HN combination significantly enhanced both FX accumulation and biomass production, leading to a higher FX productivity (*p* < 0.05). Consequently, the integration of lower light intensity and high initial nitrogen concentration yielded a maximum FX productivity of nearly 9.82 ± 0.56 mg/L/day. Figure 6b depicts the variation in the maximum FX content and productivity obtained from *P. tricornutum* cultured under different light and nitrogen combinations. HL+HN and LL+NN treatments resulted in similar FX content, whereas LL+HN enhanced by approximately 1.5 folds. The noteworthy result revealed that the integrated approach yielded FX levels 2.78-fold and 1.73-fold higher than those achieved with the single-factor application of LL and HN, respectively.

## 3. Discussion

*P. tricornutum* was cultured at different initial nitrogen levels, including LN, NN, and HN, with limited, normal, and elevated nitrate concentrations, respectively. Consistent with previous studies, the results indicated that nitrogen availability considerably influenced the physiological characteristics, including growth performance, biomass, and photosynthetic pigment production (Figure 1). Previously, the growth of *Conticribra weissflogii* and *P. tricornutum* was suppressed in the media without nitrogen supply [28,29], while *Nitzchia* sp. biomass was greatly reduced under limited nitrogen conditions [24]. On the contrary, the highest dry biomass of *C. weissfloggii* (0.75 g/L) and *Nitzchia* sp. (0.6 g/L) were attained by doubling nitrogen concentration in the culture media [24,28]. Under nitrogen-repleted conditions, *P. tricornutum* produced approximately 2.7 times higher biomass productivity than that under nitrogen-depleted conditions [30]. Nitrogen and carbon are fundamental elements required for microalgal growth and the biochemical compositions of microalgal biomass. Under autotrophic mode, carbon can be fixed via photosynthesis, while microalgae must import nitrogen from an external source, mainly sodium nitrate, dissolved in the culture media. Nitrogen limitation is associated with lower growth due to oxidative stress, lower photosynthetic efficiency, the accumulation of energy-rich compounds, and the degradation of nitrogen-containing compounds, such as proteins, DNA, and chlorophylls [23,31]. Specifically, prolonged nitrogen starvation is indicated to induce the increased accumulation of reactive oxygen species (ROS), which play a critical role in signaling the unfavorable environment [32,33]. ROS accumulation could be related to reduced photosynthetic efficiency, imbalance of the electronic transport chain, and lipid peroxidation under nitrogen starvation [33]. The activation of antioxidant enzymes, such as catalase, peroxide dismutase, and superoxide dismutase, is required to conquer nitrogen stress [33,34]. Once the excessive accumulation of ROS overwhelms the antioxidant activity, oxidative stress would lead to detrimental effects on the living organisms. Thus, nitrogen limitation has an adverse impact on microalgal growth and biomass production, whereas nitrogen repletion and supplementation boost microalgal growth and biomass production [22,28,29,35].

Nevertheless, it has also indicated that the relationship between nitrogen availability and microalgal growth is unlikely to be strictly linear. Afonso et al. [27] demonstrated accelerated *P. tricornutum* growth with increasing nitrate concentrations up to 8.82 mM; however, excessively high nitrate concentrations hindered *P. tricornutum* growth. Similarly, *I. galbana* microalga cultured with different nitrogen concentrations (0, 36, 72, 144, and 288 mg/L) achieved the highest cell density at 144 mg/L and the lowest cell density at 0 mg/L. Thus, 8.82 mM was selected as the highest concentration of sodium nitrate to prevent the inhibition of cell growth, according to Afonso’s study [27].

Furthermore, the growth performance was associated with the amount of nitrogen available in the environment (Figure 1a). Notably, nitrogen variation results in different growth behavior compared to other factors, such as light and temperature [16,36]. For example, higher light intensity can rapidly accelerate the growth rate and increase biomass compared to lower light intensity [17]. In the present study, other environmental factors were fixed to exclude their effects on *P. tricornutum*. During the early exponential phase, the specific growth rates were comparable across three nitrogen conditions. From day 4 onwards, LN samples likely entered the stationary phase when external nitrogen sources began to run out. NN and HN did not show significant differences until day 6, when nitrogen was depleted in NN samples. This concurrence was also observed in previous findings [37,38], deducing the significance of nitrogen supply in sustaining microalgal growth. Therefore, while increasing the nitrogen concentration in the culture medium alone may not accelerate the cell division rate, it is likely to prolong the duration of the exponential growth phase. As a result, this extension leads to higher cell concentration and biomass over long-term cultivation [16,39].

*P. tricornutum* is studied extensively for its rapid acclimation to new environments, particularly light acclimation [40,41]. In response to changes in external nitrogen, this species also exhibits impressive adaptability. In batch culture, the nitrogen concentration in the medium gradually decreased, indicating that the external nitrogen was transported into the microalgal cells (Figure 1a). On day 5, when nitrogen was depleted in LN, trace amounts were left in NN, and high concentrations remained in HN, which were subjected to transcriptomic analysis. The regulation of genes involved in nitrogen assimilation and uptake may directly reflect the intracellular nitrogen states of starvation, transition, and repletion in day 5 LN, NN, and HN samples, respectively. The majority of genes involved in nitrogen assimilation and uptake were downregulated in the nitrogen-replete state. There was a non-significant difference in transcript expression between the transition and starvation states. In agreement with earlier findings, genes such as nitrate transporter (*PtNIT1, PtNRT2*), nitrate reductase (*PtNR, PtFd-NiR*), ammonium transporter (*PtAMT*), glutamate synthase (*PtGOGAT-Fd*), and glutamine synthetase (*PtGSII*) were markedly downregulated by nitrogen repletion but upregulated by nitrogen starvation [42,43]. In response to the transition of nitrogen availability, some genes, such as *PtLHCR10*, *PtAMTd*, and *PtVNRT3*, with abnormal expression patterns compared to their isogenes, were also identified (Figure 3 and Figure 4), inferring the consistency of our findings with those of an earlier study [44]. Therefore, the nitrogen concentration in the environment is considered an indicator of the intracellular nitrogen state. Accordingly, only HN prolonged nitrogen repletion until day 5, which was accompanied by promoting growth and pigment accumulation.

In agreement with the growth performance, the production of photosynthetic pigments in *P. tricronutum* was also associated with external nitrogen availability (Figure 1b). Chlorophylls *a* and *c* and FX content in *P. tricornutum* grown at LN significantly reduced from day 4 onwards, while that in *P. tricornutum* grown in NN condition markedly reduced from day 6 onwards. Under the HN condition, pigment accumulation in *P. tricornutum* remained at the same high level from day 6 onwards. In *O. aurita*, chlorophylls *a* and *c* and FX content began to decline since the nitrogen supply had been depleted [26]. By examining the effect of various nitrogen concentrations on FX production, Afonso et al. [27] indicated that an increase in initial nitrogen concentration considered resulted in the enhanced FX content in *P. tricornutum*. Relevant findings have also indicated this universal effect of nitrogen availability on FX accumulation in other microalgae, such as *Nitzchia* sp., *O. aurita*, and *C. weissflogii* species [22,24,26]. Specifically, the highest FX content (~1.5 mg/g) was obtained from *C. weissflogii* cultured in the 2 N group, which was 4.5 and 1.4 times higher than those in the 0 N and 1 N cultures, respectively [28]. *Nitzchia* sp. cultured under high nitrogen concentrations resulted in a 9-fold higher FX content than that under low nitrogen concentrations [24]. Chlorophylls are nitrogenous compounds derived from glutamates; therefore, nitrogen availability must directly influence chlorophyll accumulation. Interestingly, FX has no nitrogen atom in its molecular structure but experiences a similar impact based on nitrogen availability.

Additionally, in line with chlorophylls, the positive correlation between FX content and growth (r ≥ 0.6) was observed, inferring the concurrence of FX biosynthesis and growth under the influence of various environmental factors [17,45,46]. Fluctuation of FX content in different growth stages has been reported in *Thalassiosira weissflogii* diatoms, with the highest value obtained within the exponential phase [47]. However, despite the positive correlation, FX accumulation likely reached a flat stage earlier than growth performance and was more dependent on external nitrogen availability. In particular, common results showed that FX accumulation exhibited two distinct stages, which were referred to as P1 and P2 in this study [17,27,45,46]. At stage P1, the continuous increase in FX accumulation was consistent across various culture conditions. This phenomenon could be attributed to the shading effect resulting from a reduction in light received by the microalgal cells. As the cell number increases, the light per cell decreases, leading to higher cellular FX content [48]. We found that the extension of the FX accumulation stage (P1) resulted from an increase in nitrogen supply [49].

Despite many reports on the effect of nitrogen on FX production in microalgae, the regulation of the carotenogenesis pathway in response to various nitrogen conditions has been neglected so far. The present study was more dedicated to exploring the regulation of FX biosynthesis depending on nitrogen availability. Among the genes upregulated under nitrogen repletion, *PtPDS*, *PtPSY*, and *PtVDE* genes exhibited key roles in the FX biosynthetic pathway according to previous studies related to gene function. Specifically, the overexpression of *PSY* increased the FX content up to 1.8 folds in *P. tricornutum* [12]. The silencing of *PDS* resulted in the downregulation of genes involved in subsequent steps of carotenogenesis and significantly decreased carotenoid content [50,51]. On the contrary, the introduction of foreign *PDS* from *C. reinhardtii* increased up to 2-fold higher violaxanthin content, an important precursor of FX biosynthesis [52]. Interestingly, *PtVDE* was upregulated under both nitrogen repletion and long-term nitrogen deprivation (Table S3) compared to the NN samples in the transition state. The *PtVDE* gene is a crucial gene involved in the conversion of violaxanthin to zeaxanthin and diadinoxanthin to diatoxanthin. Additionally, *PDS*, *PSY*, and *VDE* genes were highly upregulated during the growth phase, where nitrogen might be replete [45].

It has been suggested that the enhanced FX accumulation resulting from the increase in nitrogen can be attributed to the upregulation of chlorophyll biosynthesis [29]. Under nitrogen limitation, cells start to use cellular nitrogen derived from the degradation of protein complexes, such as the photosynthetic apparatus. When *P. tricornutum* experiences nitrogen depletion, the majority of key proteins responsible for light harvesting and electron transport, such as photosystem I/II, ATP synthase, and FCP, are considerably downregulated [30,53]. Accordingly, the impact of nitrogen availability on FX accumulation could be associated with FCP abundance owing to the incorporation of chlorophylls *a* and *c* and FX into the FCP complexes and the interdependence of FX biosynthesis with FCP formation [17,54]. In the present study, liquid chromatography–mass spectrometry (LC-MS) results indicated that the increment in nitrogen concentration resulted in more FCP antenna protein in the isolated thylakoid membrane (Table 1, Figure S2). Consistently, transcriptome analysis revealed the upregulation of most FCP-encoding genes under nitrogen repletion and their downregulation in response to nitrogen depletion. A similar effect of nitrogen on *LHCF* and *LHCR* families has been reported in *T. lutea* [55]. When *P. tricornutum* experiences nitrogen deprivation, FCP-encoding genes, such as *LHCR13*, *LHCF17*, *LHCF11*, *LHCF8*, and *LHCF1,* are downregulated [31,56]. Notably, compared to genes involved in carotenogenesis, *LHC* genes exhibited a co-expression pattern and a greater change in their transcript levels (Figure 4, Tables S2 and S3), indicating a more profound impact of nitrogen availability on FCP biosynthesis. Thus, nitrogen supply is pivotal for the biosynthesis of antenna proteins and the core compositions of FCP. Additionally, the highest total membrane protein content was obtained from HN samples, followed by NN samples, whereas LN samples had the lowest total membrane protein concentration. This result also suggests that *P. tricornutum* experiencing nitrogen depletion might have smaller thylakoid membranes, rather than a larger size of LHC or a higher density of LHC in microalga cells experiencing light turbulence [17]. As previously mentioned, FCP plays a crucial role in photosynthesis as peripheral light-harvesting complexes. While *P. tricornutum* growth was accelerating during the early exponential phase, a shading effect could signal the microalgal cells to require more FCP to receive more light. After the exponential phase, the growth was likely inhibited by nutrient depletion, light per cell was assumed to be constant. Notably, the FX content was significantly reduced in stage P2, associated with a nitrogen-depleted state, whereas nitrogen repletion likely maintained a high level of FX. This could be attributed to the association between FCP abundance and nitrogen availability.

The enhancement of both FX biosynthesis and biomass production is required to enhance FX productivity. Lower light intensity and nitrogen repletion are two culture factors playing pivotal roles in FX production [16,17]. Lower light intensity always reduces growth but profoundly increases FX biosynthesis in *P. tricornutum* [16,17,57]. In contrast, nitrogen repletion elongated the exponential phase and prolonged the FX accumulation stage (P1). HN was able to increase biomass and reverse the negative effect of higher light intensity on FX accumulation, leading to not only an equal FX content between HL+HN and LL+NN treatments but also markedly higher FX productivity (Figure 6b). Still, the highest FX productivity was obtained under the LL+HN group. Particularly, biomass production continuously elevated throughout the cultivation period, while stage P1 of FX accumulation was also prolonged for 10 day, which was much longer than that under HL+HN and LL+NN conditions (6 day). Therefore, our results highlight the potential of light and nitrogen combinations to maximize FX productivity, in which the combination of low light and high nitrogen could be an effective strategy for enhancing FX productivity from batch cultures of *P. tricornutum*.

## 4. Materials and Methods

### 4.1. Chemicals

Sea salt (Red Sea Salt) was purchased from Red Sea Fish Pharm. Ltd. (Eilat, Israel). Sodium nitrate was obtained from Daejung Chemical & Metals (Siheung, Republic of Korea). Organic solvents, including absolute ethanol, acetonitrile (ACN), and methanol, were obtained from Fisher Scientific (Pittsburgh, PA, USA). Bovine serum albumin was obtained from Thermo Fisher Scientific (Waltham, MA, USA). Ethylenediaminetetraacetic acid disodium salt dehydrate (Na_2_EDTA), magnesium chloride hexahydrate (MgCl_2_·6H_2_O), manganese(II) chloride tetrahydrate (MnCl_2_·4H_2_O), 4-(2-Hydroxyethyl)piperazine-1-ethanesulfonic acid, N-(2-Hydroxyethyl)piperazine-N′-(2-ethanesulfonic acid) (HEPES), potassium chloride (KCl), sorbitol, and 1,4-dithiothreitol (DTT) were purchased from Sigma-Aldrich (Saint Louis, MO, USA). The FX standard was purchased from Sigma-Aldrich.

### 4.2. P. tricornutum Cultivation

*P. tricornutum* UTEX 646 Bohlin strain 4 (Pt4, UTEX 646) was obtained from the University of Texas (https://utex.org/ accessed on 1 September 2019). *P. tricornutum* starter was maintained in artificial seawater supplemented with f/2 medium compositions under continuous illumination at a light intensity of ~70 µmol photons/m^2^/s. In this study, *P. tricornutum* microalgae were cultured at three different initial nitrogen levels, including 0.22 mM sodium nitrate (LN), 0.882 mM sodium nitrate (NN), and 8.82 mM sodium nitrate (HN), for 10 days. LN, NN, and HN refer to f/2 media with initially limited nitrogen, normal nitrogen, and highly elevated nitrogen levels, respectively. *P. tricornutum* starter was inoculated into a 2 L medium enriched with f/2 compositions at an initial density of 1 × 10^5^ cells/mL. The culture conditions were consistent with starter maintenance and maintained throughout the cultivation period; essentially, the temperature, light intensity, and aeration rate were set at 20 ± 2 °C, ~70 µmol photons/m^2^/s, and ~10 cm^3^/s, respectively. Three biological replicates were used for each group.

In the latter experiment, *P. tricornutum* was cultured under various light and nitrogen combinations, encompassing low light and high initial nitrogen (LL+HN), low light and normal nitrogen (LN+NN), and high light and high initial nitrogen (HL+HN) for 14 days. HL and LL were set at 100 and 20 µmol/m^2^/s, respectively. Cell number, biomass, FX content, and FX productivity were determined every two days. All experiments were conducted with three biological replicates for each group.

### 4.3. Growth Determination and Biomass Production

Cell density (cells/mL) was determined using a hemocytometer. Briefly, cell density was calculated using the following equation:

$$N\;\left(\mathrm{cells}/\mathrm{mL}\right)=N_{0}\times 10^{4}$$

where N is the total cell number in 1 mL (cells/mL) and N_0_ is the actual number of cells counted with a hemocytometer. Biomass concentration was determined based on the fresh weight of at least 10 mL culture samples. After 15 min centrifugation at 3000× *g* and 4 °C, the supernatant was discarded, while the cell pellet was resuspended with distilled water and transferred into a pre-weighted microtube. Washing the pellets was performed with a short centrifugation at 8000× *g*, and 4 °C. Water-free fresh biomass was weighed with an analytical balance with a standard deviation of ±0.1 mg. Fresh samples were stored at −80 °C for further pigment analyses. Biomass concentration was calculated using the following equation:

$$\mathrm{Biomass}\;\mathrm{concentration}\;\left(g/L\right)=\frac{m}{V}$$

where m and V represent fresh biomass and the actual amount of culture collected for the measurement of microalgal biomass, respectively.

The specific growth rate (µ) was calculated with the following equation [58]:

$$\mu =(\ln N_{i\;}-\ln N_{0})∕\left(t_{i}-t_{0}\right)$$

where N_0_ and N_i_ represent the microalgal biomass at t_0_ and t_i_ days, respectively.

### 4.4. Quantification of Primary Photosynthetic Pigments

#### 4.4.1. Fucoxanthin Quantification with HPLC-UV-DAD Analysis

The methodology used for FX quantification has been described in our previous study with minor modifications [17]. Briefly, FX extract was prepared and injected into an analytical vial for high-performance liquid chromatography coupled with diode array detector (HPLC-DAD) analysis. A volume of a 10 μL sample was injected into a YMC-Pack ODS-A column (250 × 4.6 mm I.D). The flow rate and column temperature were maintained at 0.8 mL/min and 30 °C, respectively. Binary mobile phases consisting of 0.1% formic acid in ACN (A) and 0.1% formic acid in water (B) were employed, following a gradient program at 0 min (85:15, A:B), 20 min (100:0, A:B), and 25 min (100:0, A:B). FX standard solutions at 3.125, 6.25, 12.5, 25, 50, and 100 µg/mL were prepared for the construction of the standard curve.

#### 4.4.2. Quantification of Chlorophylls with a UV/Vis Spectrophotometer

Chlorophylls *a* and *c* were determined from the same extract prepared for FX analysis using the method described in our previous study [17]. Briefly, the optical density of the extract was measured using a Cary 60 UV-Vis spectrophotometer at wavelengths of 632 nm and 665 nm. The concentrations of chlorophylls *a* and *c* in diatoms were calculated according to the following Ritchie’s equations (2006) [59]:

$$Chl\;a\;\left(\mu g/\mathrm{mL}\right)=13.2654\times A_{665\mathrm{nm}}-2.6839\times A_{632\mathrm{nm}}\;Chl\;c\left(\mu g∕\mathrm{mL}\right)=28.8191\times A_{632\mathrm{nm}}-6.0138\times A_{665\mathrm{nm}}$$

### 4.5. Total Nitrogen Content in P. tricornutum Culture Medium

The relative nitrogen concentration available in the culture medium was determined by adopting the optical method described elsewhere [39]. Briefly, 2 mL of *P. tricornutum* grown at different initial nitrogen levels was collected every two days. After centrifugation at 12,000× *g* and 4 °C for 10 min, the supernatant was collected by filtration with a 0.45 µm pore size membrane. The absorbance of culture samples and standard mixture at wavelengths of 220 nm and 275 nm was determined using a Lamda35 UV/Vis spectrophotometer (PerkinElmer, Waltham, MA, USA). Sodium nitrate solutions of at least four different concentrations (6.25, 12.5, 25, and 50 mg/L) were prepared to construct a standard curve. The difference between optical density at 220 nm and 275 nm was used to calculate the relative amount of nitrogen in the medium.

### 4.6. Semi-Quantification of Fucoxanthin–Chlorophyll a/c-Binding Proteins

#### 4.6.1. Thylakoid Membrane Isolation

Thylakoid membrane fractions were isolated from samples collected on day 5 by adopting the method described by Levitan et al. [60], with minor modifications. Briefly, the cell pellet was first suspended in 3 mL breaking buffer A (2 mM Na_2_EDTA, 1 mM MgCl_2_·6H_2_O, 1 mM MnCl_2_·4H_2_O, 50 mM HEPES (pH 7.5), and 0.66 M Sorbitol). Then, the cell wall was broken using an ultrasonicator (20 s:20 s, three cycles) at an amplitude of 30 Hz. Cell debris and unbroken cells were removed by centrifuging at 3000× *g* for 8 min. The debris-free supernatant was carefully loaded onto a 2 M sucrose cushion and centrifuged at 16,000× *g* for 15 min. The upper layer was collected into a new microtube. The thylakoid membrane fraction was precipitated with 1 mL of buffer B (6 mM Na_2_EDTA, 1 mM MnCl_2_·4H_2_O, 5 mM MgCl_2_·6H_2_O, 50 mM HEPES (pH 7.5), and 10 mM KCl) by centrifuging at 20,000× *g* for 15 min. Subsequently, the collected fraction was washed twice with 1.5 mL buffer C (5 mM Na_2_EDTA, 10 mM HEPES (pH 7.5), and 0.2 M KCl) and buffer D (5 mM MgCl_2_·6H_2_O, and 50 mM HEPES (pH 7.5)). The isolated thylakoid membrane fraction was dissolved into a small amount of phosphate buffer saline (PBS) and stored at −80 °C for further analyses, including FCP proteomic and the quantification of photosynthetic pigments. All the steps were conducted on ice under dim light.

#### 4.6.2. Proteomic Analysis with SDS-PAGE Coupled with LC-MS Analysis

Semi-quantification of FCP proteins was conducted as previously described [17]. Briefly, a mixture containing 20 µg membrane protein, DTT 50 mM, and Laemmil sample buffer 1X was heated at 100 °C for 3 min to denature the protein. Protein samples were loaded into 4–12% NuPAGE Bis–Tris gels (Life Technologies, Carlsbad, CA, USA), followed by staining with 0.1% Coomassie Brilliant Blue reagent. The target protein, located between 15 and 18 kD, was excised into 1mm^3^ cubes and digested with trypsin according to the protocol of Shevchenko et al. (2006) [61] and Kang et al. (2014) [62]. The digested peptides were concentrated using SpeedVac (SPD1030, Thermo Fisher Scientific), reconstituted in 10 μL of solvent A (0.1% formic acid in water), and centrifuged. FCP proteomic analysis was performed using an LC-MS/MS equipped with an Easy-nLC 1000 system and a Q Exactive Orbitrap high-resolution mass spectrometer (Thermo Fisher Scientific). All MS/MS samples were analyzed using SEQUEST in Proteome Discoverer (Thermo Fisher Scientific; version 2.4.1.15). SEQUEST was set up to search against a highly annotated protein database of *P. tricornutum* downloaded from UniProt.org on 6 June 2021, with a total of 556,484 entries. Scaffold (version Scaffold_5.2.1, Proteome Software Inc., Portland, OR, USA) was used to validate MS/MS-based peptide and protein identifications. Peptide identifications were accepted if they could be established at a probability greater than 95.0% by the Scaffold Local FDR algorithm. Protein identifications were accepted if they could be established at a probability greater than 99.0% to achieve an FDR of less than 1.0% and contained at least two identified peptides.

### 4.7. RNA Sequencing Analysis of Day 5 Samples

Total RNA was isolated from LN, NN, and HN samples collected on day 5 using the method developed within our laboratory. Briefly, total RNA was extracted from cell pellets using 1 mL of RNAiso plus reagent (TaRaKa, Osaka, Japan) according to the manufacturer’s instructions. Then, RNA samples were purified using a Plant RNeasy Mini kit (Qiagen, Valencia, CA, USA) included with DNase treatment. Quality control analyses of RNA concentration, purity, and integrity were performed to obtain qualified mRNA samples. Quality control, library construction, and sequencing were performed according to the standard protocol available at Seeders Inc., South Korea (http://www.seeders.co.kr/ accessed on 1 March 2023). Next-generation sequencing was processed using an Illumina HISeq X-based paired-end. Mapping and RNA-seq data analysis were performed with a pipeline adopted elsewhere. Briefly, gene mapping to the reference genome of *P. tricornutum* (ASM15095v2) obtained from Ensembl Protist (https://protists.ensembl.org/index.html accessed on 1 June 2023) was conducted with the STAR tool. BAM files were obtained and used for differentially expressed gene analysis using DESeq2 in the Bioconductor package installed in R version 4.3.1. RNA-seq data retrieval and visualization were performed using R software. The enrichment of gene ontology and KEGG pathways was investigated using an online bioinformatics tool called ShinyGO 0.76 (http://bioinformatics.sdstate.edu/go/ accessed on 15 August 2023) developed by South Dakota State University.

### 4.8. Statistical Analysis

All data are presented as the mean value ± standard deviation of at least three replicates. For statistical analysis, one-way ANOVA followed by Tukey’s test at a significance level of *p* < 0.05, were performed using GraphPad Prism 7 (GraphPad Software Inc., San Diego, CA, USA).

## 5. Conclusions

In summary, *P. tricornutum* growth, biomass production, and photosynthetic pigment accumulation are strongly associated with external nitrogen availability. Our results highlighted the crucial role of nitrogen availability as a limiting factor for FX production. Elevating the initial nitrogen concentration increased FX productivity by elongating the exponential phase, leading to higher biomass production and prolonging stage P1, which is associated with a continuous increase in FX biosynthesis. The increase in FX biosynthesis resulted from not only the upregulation of key genes involving carotenogenesis and regulating rate-limiting steps toward FX biosynthesis, such as *PtPSY*, *PtPDS*, and *PtVDE,* under nitrogen repletion but also from higher FCP abundance in the thylakoid membrane. This is critical for FX biosynthesis due to the interdependence of FX and FCP. Finally, the combination of appropriate light and nitrogen could effectively maximize FX productivity by promoting both FX biosynthesis and biomass production. These findings contribute to the well-known economic prospects of using microalgae, essentially *P. tricornutum*, as alternative sources for commercial FX production.

## Figures and Tables

**Table 1:** Normalized weighted spectrum count of fucoxanthin–chlorophyll *a/c*-binding antenna proteins identified in the thylakoid membrane isolated from day 5 samples of *Phaeodactylum tricornutum* microalgae cultured at different initial nitrogen concentrations [low nitrogen (LN), normal nitrogen (NN), and high nitrogen (HN)].

Accession No.	Alternate ID	LN	NN	HN
B7FRW5	LHCF1	2857 ± 273 ^c^	11,243 ± 1525 ^b^	21,399 ± 1584 ^a^
B7FRW4	LHCF2	2844 ± 328 ^c^	12,718 ± 1318 ^b^	22,923 ± 1385 ^a^
B7FRW2	LHCF3/4	13,016 ± 1422 ^c^	39,244 ± 945 ^b^	67,185 ± 3149 ^a^
B7GBK7	LHCF5	3124 ± 433 ^c^	16,442 ± 2394 ^b^	31,459 ± 1594 ^a^
B7G5S7	LHCF6	2270 ± 267 ^c^	7624 ± 1040 ^b^	15,851 ± 1660 ^a^
B7G6Y1	LHCF8	5580 ± 1122 ^c^	18,586 ± 1326 ^b^	32,556 ± 2839 ^a^
B7G955	LHCF9	401 ± 69 ^c^	1230 ± 399 ^b^	2804 ± 1421 ^a^
B7G5B6	LHCF10	3297 ± 718 ^c^	14,299 ± 1525 ^b^	26,277 ± 1850 ^a^
B7GBK6	LHCF11	4659 ± 1123 ^c^	15,459 ± 669 ^b^	25,301 ± 1245 ^a^
B7GCA2	LHCF12	3484 ± 984 ^c^	11,945 ± 791 ^b^	19,205 ± 1901 ^a^
B7G871	LHCF13	254 ± 46 ^c^	843 ± 279 ^b^	1646 ± 317 ^a^
B5Y5L4	LHCF14	1415 ± 231 ^c^	4813 ± 580 ^b^	9206 ± 460 ^a^
B7FV42	LHCF16	80 ± 0 ^c^	351 ± 161 ^b^	610 ± 381 ^a^
B7GC99	LHCF17	334 ± 61 ^c^	1124 ± 219 ^b^	1890 ± 761 ^a^
B7FUM6	LHCR1	587 ± 101 ^c^	1124 ± 122 ^b^	1585 ± 211 ^a^
B7FSP4	LHCR3	935 ± 23 ^c^	2846 ± 105 ^b^	4938 ± 317 ^a^
B7FQE1	LHCR4	267 ± 23 ^c^	1230 ± 61 ^b^	1890 ± 381 ^a^
B7FQE0	LHCR12	1695 ± 311 ^c^	5059 ± 422 ^b^	7682 ± 1018 ^a^
B7G502	LHCR13	854 ± 46 ^c^	2846 ± 658 ^b^	4694 ± 1598 ^a^
B7G503	LHCR14	1268 ± 228 ^c^	5973 ± 896 ^b^	9511 ± 2420 ^a^
B7FYL0	LHCX1	240 ± 40 ^c^	773 ± 265 ^b^	1280 ± 484 ^a^

Different letters denote the significant difference amongst three groups analyzed with one-way ANOVA followed by Tukey’s test, *p* < 0.05.

## Data Availability

Raw sequencing reads obtained from RNA sequencing in this study have been deposited in the National Center for Biotechnology Information sequence read archive. Data can be found at the following link: https://www.ncbi.nlm.nih.gov/bioproject/PRJNA1002804.

## Supplementary Materials

The following supporting information can be downloaded at https://www.mdpi.com/article/10.3390/md22020093/s1, Figure S1: A correlation analysis of observed traits, including cell density, biomass, chlorophyll *a* and *c*, and fucoxanthin production of *Phaeodactylum tricornutum* microalgae cultured at different initial nitrogen concentrations [limited nitrogen (LN), normal nitrogen (NN), and high nitrogen (HN)], *p* < 0.05; Figure S2: (a) Chlorophyll *a* and *c* and (b) fucoxanthin content of isolated thylakoid membrane from day 5 samples of *Phaeodactylum tricornutum* microalgae cultured at different nitrogen conditions [limited nitrogen (LN), normal nitrogen (NN), and high nitrogen (HN)]. Different letters denote significant differences amongst the three groups analyzed with one-way ANOVA, *p* < 0.5. FX, fucoxanthin; TM, thylakoid membrane; Figure S3: Top gene ontology (GO) enrichment of genes found in clusters I and V. The analysis was performed using an online informatics tool, ShinyGO, with a cutoff of 0.05; Figure S4: The top Kyoto Encyclopedia of Genes and Genomes (KEGG) pathways of genes found in clusters I and V. The analysis was performed using an online informatics tool, ShinyGO, with a cutoff < 0.05; Table S1: Information of identified genes involved in nitrogen uptake and assimilation in *Phaeodactylum tricornutum*; Table S2: Information of identified genes involved in fucoxanthin–chlorophyll *a/c*-binding protein biosynthesis in *Phaeodactylum tricornutum*; Table S3: Information of identified genes involved in methylerythritol 4-phosphate and carotenogenesis pathways of fucoxanthin biosynthesis in *Phaeodactylum tricornutum.*
