# Supplementary material for: Understanding the Impact of Nitrogen Availability: A Limiting Factor for Enhancing Fucoxanthin Productivity in Microalgae Cultivation
Source: Mar Drugs. 2024 Feb 18;22(2):93. doi: 10.3390/md22020093 (PMC10889934; doi:10.3390/md22020093)
Supplement: Supplementary file 1 [file marinedrugs-22-00093-s001.zip › marinedrugs-2860403 supplementary materials.pdf]

Supplementary Materials

Understanding the impact of nitrogen availability: a limiting factor for enhancing fucoxanthin productivity in microalgae cultivation

To Quyen Truong[1,2], Yun Ji Park[2], Jessica Winarto[1,3], Phuong Kim Huynh[1,2], Jinyoung Moon[2], Yeong Bin Choi[2], Dae-Geun Song[3], Song Yi Koo[3], Sang Min Kim[1,2*]

[1] Division of Bio-Medical Science & Technology, Korea Institute of Science and Technology (KIST) School, University of Science and Technology, Seoul 02792, Republic of Korea; ttquyen_vn@kist.re.kr (T.Q.T.); jessicawinarto@kist.re.kr (J.W.); 623024@kist.re.kr (P.K.H.)

[2] Smart Farm Research Center, KIST Gangneung Institute of Natural Products, Gangneung 25451, Republic of Korea; pyj@kist.re.kr (Y.J.P.); jy77mun@kist.re.kr (J.M.); choiyeongbin@kist.re.kr (Y.B.C.)

[3] Natural Product Informatics Research Center, KIST Gangneung Institute of Natural Products, Gangneung 25451, Republic of Korea; dsong82@kist.re.kr (D.-G.S.); ninesong2@kist.re.kr (S.Y.K.)

* Correspondence: kimsm@kist.re.kr; Tel.: +82-33-650-3640; Fax: +82-33-650-3679

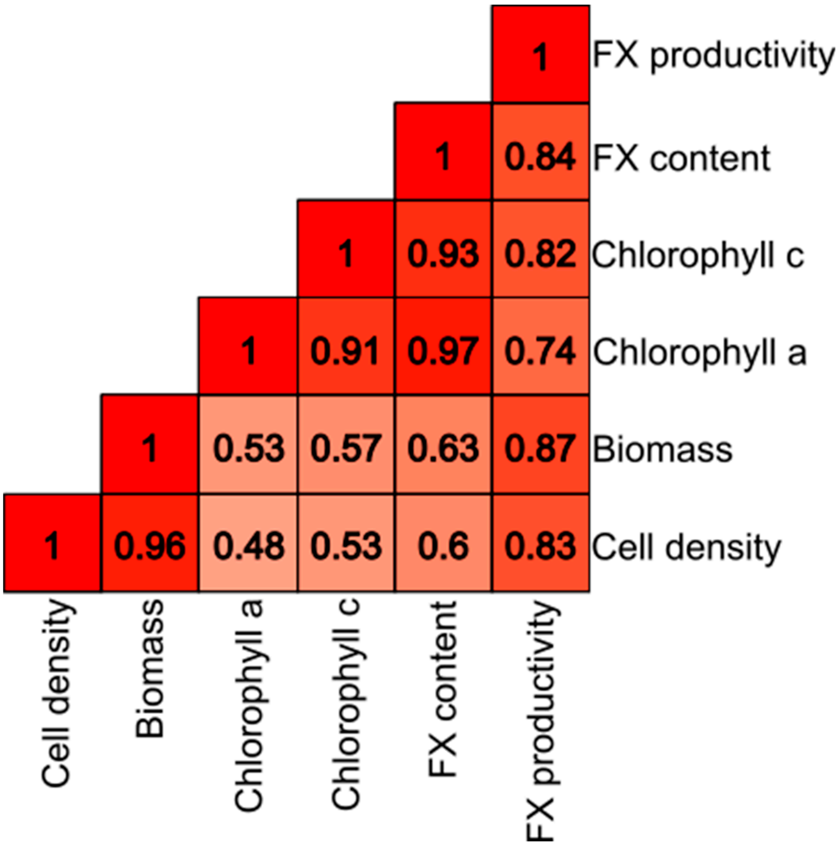

Supplementary Figure S1. The correlation analysis of observed traits including cell density, biomass, chlorophyll *a*, *c*, and fucoxanthin production of *Phaeodactylum tricornutum* microalgae cultured at different initial nitrogen concentrations [limited nitrogen (LN), normal nitrogen (NN), and high nitrogen (HN)], $p < 0.05$.

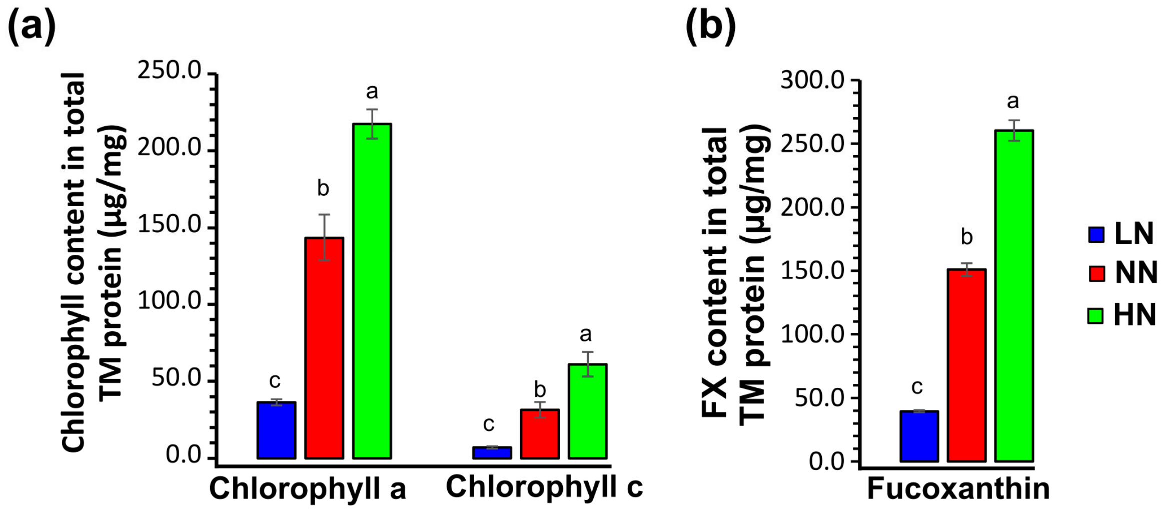

Supplementary Figure S2. (a) Chlorophyll *a* and *c* and (b) fucoxanthin content of isolated thylakoid membrane from day-5 samples of *Phaeodactylum tricornutum* microalgae cultured at different nitrogen conditions [limited nitrogen (LN), normal nitrogen (NN), and high nitrogen (HN)]. Different letters denote significant difference amongst three groups analyzed with One-way ANOVA test, $p < 0.5$. FX, fucoxanthin; TM, thylakoid membrane.

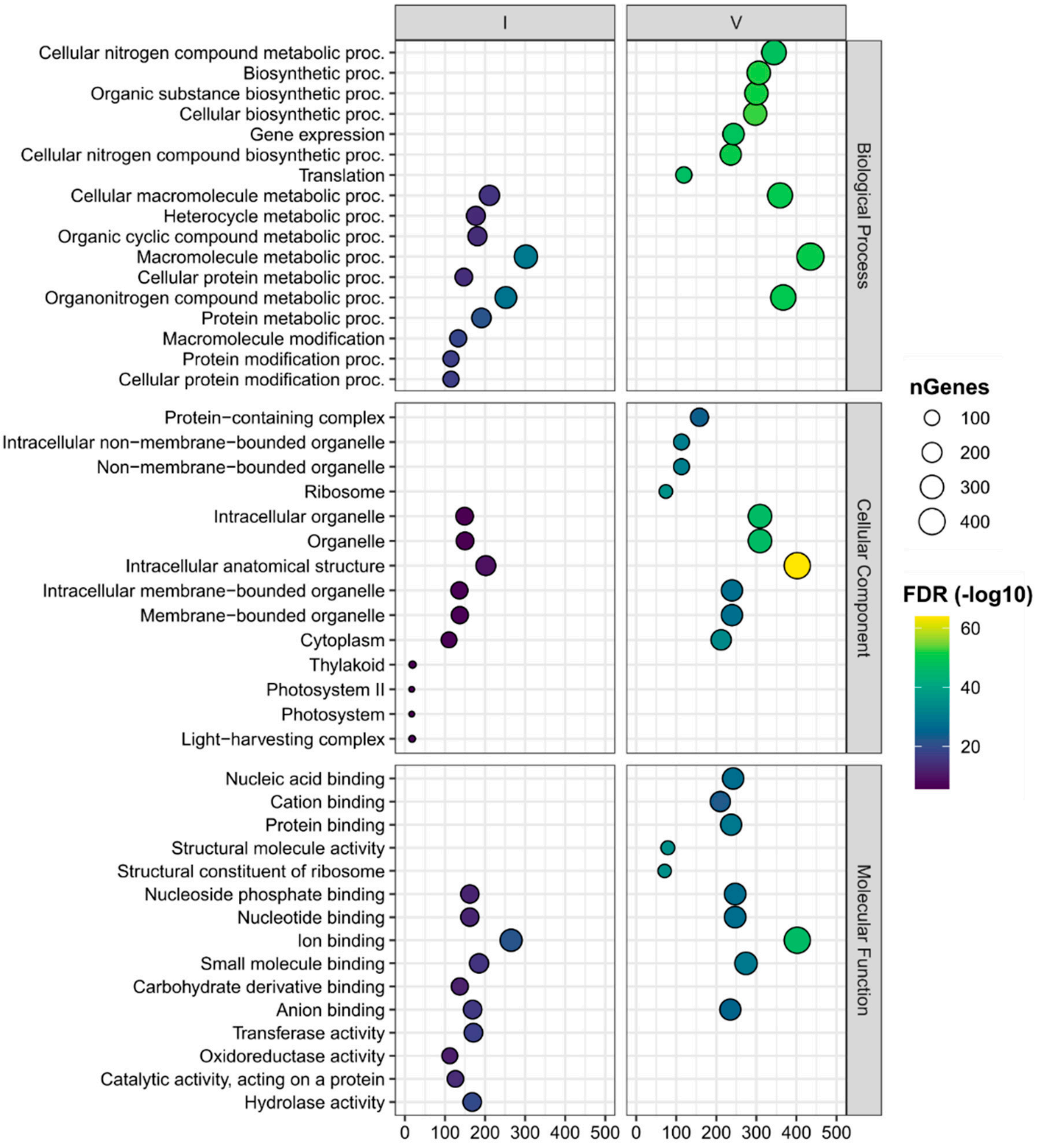

Supplementary Figure S3. Top Gene Ontology enrichment of genes found in Cluster I and V. The analysis was performed using online informatics tool-ShinyGO with cutoff 0.05.

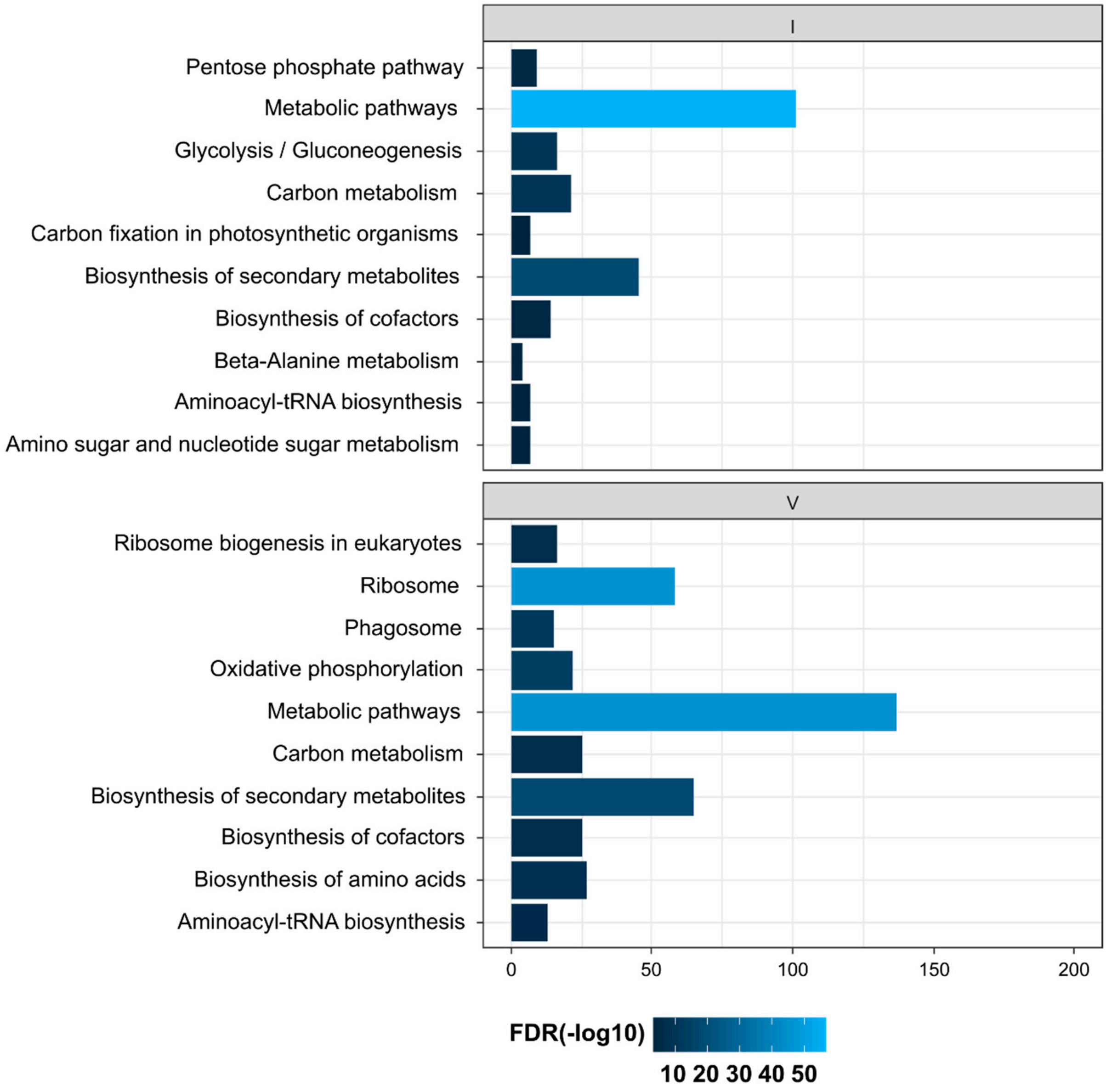

Supplementary Figure S4. Top Kyoto Encyclopedia of Genes and Genomes pathways of genes found in Cluster I and V. The analysis was performed using online informatics tool-ShinyGO with a cutoff < 0.05.
